# EP2 receptor mediated cAMP release is augmented by PGF_2α_ activation of the FP receptor via the calcium-calmodulin pathway

**DOI:** 10.1016/j.cellsig.2009.09.012

**Published:** 2010-01

**Authors:** A.B. Abera, K.J. Sales, R.D. Catalano, A.A. Katz, H.N. Jabbour

**Affiliations:** a MRC Human Reproductive Sciences Unit, The Queen's Medical Research Institute, 47 Little France Crescent, Edinburgh EH16 4TJ, UK; b MRC/UCT Research Group for Receptor Biology, Institute of Infectious Disease and Molecular Medicine and Division of Medical Biochemistry, Faculty of Health Sciences, University of Cape Town, Cape Town, South Africa

**Keywords:** COX, cyclooxygenase, DMEM, Dulbecco's modified eagle medium, DMSO, dimethylsulphoxide, EGFR, epidermal growth factor receptor, ERK, extracellular signal-regulated kinase, GPCR, G protein-coupled receptor, IBMX, 3-isobutyl-1-methyl xanthine, PBS, phosphate buffered saline, RT, reverse transcriptase, SAT1, Spermidine/N1-acetyltransferase, siRNA, small interfering RNA, VEGF, vascular endothelial growth factor, Prostaglandin, Adenylyl cyclase, cAMP, SAT1

## Abstract

Prostaglandins exert their effects on target cells by coupling to specific G protein-coupled receptors (GPCRs) that are often co-expressed in the same cells and use alternate and in some cases opposing intracellular signaling pathways. This study investigated the cross-talk that influences intracellular signaling and gene expression profiling in response to co-activation of the EP2 and FP prostanoid receptors in Ishikawa cells stably expressing both receptors (FPEP2 cells). In this study we show that in FPEP2 cells, PGF alone does not alter adenosine 3′,5′-cyclic monophosphate (cAMP) production, but in combination with Butaprost enhances EP2 receptor mediated cAMP release compared to treatment with Butaprost alone. PGF-mediated potentiation of cAMP release was abolished by antagonism of the FP receptor, inhibition of phospholipase C (PLC) and inositol phosphate receptor (IP3R) whereas inhibition of protein kinase C (PKC) had no effect. Moreover, inhibition of calcium effectors using calmodulin antagonist (W7) or Ca^2+^/calmodulin-dependent kinase II (CaMK-II) inhibitor (KN-93) abolished PGF potentiation of Butaprost-mediated cAMP release. Using siRNA molecules targeted against the adenylyl cyclase 3 (AC3) isoform, we show that AC3 is responsible for the cross-talk between the FP and EP2 receptors. Using gene array studies we have identified a candidate gene, Spermidine/N1-acetyltransferase (SAT1), which is regulated by this cAMP mediated cross-talk. In conclusion, this study demonstrates that co-activation of the FP and EP2 receptors results in enhanced release of cAMP via FP receptor-Gα_q_-Ca^2+^-calmodulin pathway by activating calcium sensitive AC3 isoform.

## 1 Introduction

Prostaglandins exert paracrine and autocrine effects on target cells by coupling to their specific GPCRs and activate intracellular signaling. PGE_2_ and PGF_2α_ (PGF) are the most abundantly biosynthesized prostaglandins and are major metabolites of cyclooxygenase (COX) enzymes in the human endometrium [1,2]. Furthermore, COX enzyme expression, prostaglandins synthesis and their cognate receptors expression (mainly EP2 and FP) are dysregulated in endometrial adenocarcinoma [3–5].

EP2 and FP receptors are often co-expressed in the same cells and use different intracellular signaling pathways. EP2 receptors couple to Gα_s,_ resulting in increased formation of cAMP, while FP receptors couple to Gα_q_ which in turn results in release of inositol-1,4,5-triphosphate (IP) and dialcylglycerol (DAG) [1,6–8]. cAMP is generated in cells from adenosine triphosphate (ATP) by the enzymatic activity of the adenylyl cyclase (AC) family members. There are multiple AC isoforms (9 membrane-bound and 1 soluble) with different amino acid sequence, tissue distribution and regulation. AC isoforms are named from AC1 to AC9 in the order of their publication [9,10]. Difference in regulation of these isoforms includes sensitivity to calcium, G proteins (Gα_i_ and G_βγ_), protein kinases (PKA, PKC, calmodulin and calmodulin-kinase) and phosphatases (calcineurin). Out of the ten AC isoforms three (AC1, AC3 and AC8) are calcium stimulated while AC5 and AC6 are normally inhibited by calcium *in vivo* [10,11]. The intracellular calcium pathway has been shown to play a significant part in Gα_s_–Gα_q_ cross-talk by regulating calcium sensitive AC isoforms [12–14]. For example, Ostrom et al. [13] showed a potentiation of β-adrenergic (β-AR) receptor induced cAMP production by activation of angiotensin II (ANG-II) receptor coupled to Gα_q_ protein through calcium/calmodulin pathway demonstrating cross-talk between ANG-II and β-AR receptors in cardiac fibroblasts cells.

GPCR cross-talk in the eicosaniod family has been shown in several studies. Walsh and Kinsella [15] showed a cross-talk between thromboxane A_2_ (TPα and TPβ) and EP1 receptors in HEK 293 cells that leads to desensitization and inhibition of signaling of the TP receptors in a PKC-dependent manner. The same group further indentified that the activation of the FP receptor by PGF can also mediate desensitization of the TPα and TPβ receptors via the PKC pathway [16]. In another study, Wilson et al. [17] showed that heterodimerzation can occur between the human receptors for prostacyclin (IP) and TPα that leads to augmentation of TPα receptors-mediated accumulation of cAMP by IP receptors when they are co-expressed in a HEK 293 cell line.

Since EP2 and FP receptors are co-expressed in endometrial adenocarcinoma cells [7,18], this study investigated the cross-talk that may influence intracellular signaling and target gene activation in response to co-activation of EP2 and FP receptors.

## 2 Materials and methods

### 2.1 Reagents

All chemicals used were molecular biology grade and were obtained from Sigma (Dorset, UK or RSA) or IBI (Cambridge, UK). Cell culture media was purchased from Gibco (Gibco, Paisely, UK). Hygromycin (100 mg/ml stock) was purchased from Invitrogen (Invitrogen, Autogen Bioclear UK) while G418 (100 mg/ml stock in PBS), indomethcin (3 mg/ml stock in ethanol), Butaprost (5 mM stock in ethanol), PGF (100 µM stock in ethanol) and 3-isobutyl-1-methyl xanthine (IBMX, 20 mM stock in 50% ethanol) were purchased from Sigma (Sigma chemical Co., Nottingham, UK). FP receptor antagonist (AL8810, 50 mM stock in ethanol), IP3R blocker (2-APB, 40 mM stock in DMSO), PLC inhibitor (U73122, 10 mM stock in DMSO), calmodulin antagonist (W7, 25 mM stock in DMSO), CaMK-II inhibitor (KN-93, 50 mM stock in DMSO) and PKC inhibitor (Ro-31-822, 1 mM stock in DMSO) were all purchased from Calbiochem (Calbiochem, Nottingham, UK). Inhibitor of Gα_q_ (YM254890, 1 mM stock in DMSO) was a kind gift from M. Taniguchi, (Astellas Pharmaceuticals Inc., Tokyo, Japan). The EP2 and FP receptor primary antibodies were purchased from Cayman Chemical Company (Axxora, Nottingham, UK) and primary antibody for actin was purchased from Santa Cruz Biotechnology (Santa Cruz, Wiltshire, UK). Fluorescent secondary antibodies were purchased from Li-Cor Biosciences (Li-Cor Biosciences, Cambridge, UK). Stealth siRNA duplex oligoribonucleotides for AC1 and AC3 were purchased from Invitrogen (Invitrogen, Paisley, UK).

### 2.2 Cell culture

Ishikawa cells were maintained as described previously [18]. FPEP2 clones were maintained in G418 and hygromycin (200 µg/ml each) to select for the expression of FP and EP2 receptor, respectively.

### 2.3 EP2 receptor amplification and stable cell line transfection

To make stable cell lines expressing both EP2 and FP receptors, Ishikawa cells stably expressing the FP receptor (FPS32 cell lines) were used as a parental cell line [7]. [6]. cDNA from proliferative endometrium was synthesised as previously described [6] and EP2 receptor was amplified using forward 5′-TCTCTTTTCCAGGCACCCCAC-3′ and reverse 5′-TTTTAAACTGACCTCAAAGGTCAGC-3′ primers. EP2 receptor cDNA was ligated into the pcDNA3.1 (Invitrogen) expression vector in both sense and antisense directions and was transfected to the FPS32 cells using SuperFect^®^ transfection reagent (QIAGEN, UK) according to manufacturer's recommendations. Transfected cells were selected in a medium containing 800 µg/ml of hygromycin in parallel with untransfected cells. Once untransfected cells had died, hygromycin-resistant clones were picked and expanded under the selective medium. Clones were then screened for the expression of the EP2 receptor by quantitative real-time RT-PCR and for their ability to produce intracellular cAMP in response to Butaprost treatment. Three FPEP2 clones (clones 4, 8 and 10) with similar expression levels and biochemical characteristics were selected for further investigation. Data on FPEP2 clone 8 is presented here, with similar data obtained using the other two clones.

### 2.4 cDNA synthesis and real-time RT-PCR

Total RNA was extracted from Ishikawa cells using Tri-Reagent (Sigma-Aldrich Corp., Poole, UK) as manufacturer's recommendations and cDNA was synthesised from total mRNA as described previously [18]. Thereafter, quantitative real-time RT-PCR was performed with specific E and F prostanoid (EP1, EP2, EP3, EP4 and FP receptors), AC1, AC3 and SAT1 primers and probes (Table 1) as described previously [18]. Expression of analyzed genes was normalized to RNA loading for each sample using the 18S ribosomal RNA as an internal standard. Results are expressed as fold increase above vehicle treated cells. Data are presented as mean ± SEM from at least 3 independent experiments.

### 2.5 Protein extraction from cells, SDS-PAGE and western blotting

Ishikawa cells were seeded at a density of 5 × 10^5^ cells in 6 cm dishes overnight. The following day protein was extracted from the cells as described previously [18]. Total protein was quantified using standard BIO-RAD D_C_ assay (Bio-Rad, Hemel Hempstead, UK) as directed by the manufacturer's instruction. A total of 40 μg of protein was resuspended in 1× Laemmli buffer (125 mM Tris–HCL pH 6.8, 4% SDS, 20% glycerol, 5% 2-mercapthoethanol and 0.05% bromophenol blue) and boiled for 5 min at 95 °C. Samples were resolved and immunoblotted as described previously [18] prior to incubating with the rabbit anti-EP2 or FP receptor in combination with goat anti-actin primary antibody (all in 1:1000) at 4 °C overnight. The membrane was then washed with PBS-Tween and incubated with the secondary fluorescence donkey anti-rabbit IgG conjugated to Alexa Fluor 680 (Invitrogen) and donkey anti-goat IgG conjugated to IRDYE 800 (Tebu-bio, Peterborough, UK) for 1 h in the dark at 25 °C*.* After washing the membrane proteins were then viewed using Odyssey Infrared imaging system (Li-Cor Bioscience, Cambridge, UK).

### 2.6 Immunofluorescence microscopy of cells

The site of EP2 and FP receptor expression in FPS32 and FPEP2 cells were localised using immunofluorescence microscopy as described previously [19]. Briefly, 100,000 cells/well were plated out in 2-well cell chamber slides and left to adhere overnight. The next day cells were fixed with ice-cold 4% paraformaldehyde (PFA) for 20 min and washed with PBS, before being blocked by 5% BSA diluted in normal goat serum for 2 h. Thereafter, the cells were incubated with polycolonal rabbit anti-EP2 and FP receptor antibody diluted in normal goat serum (1:100) overnight at 4 °C. The next day, cells were washed three times in PBS for 10 min and incubated with Alexa Flour 488 goat anti-rabbit IgG (1:200 in PBS; Molecular Probes) for 2 h. Slides were counterstained with DAPI (1:1000 in PBS; Sigma) for 10 min for nuclear visualisation, washed and mounted in Permafluor (Immunotech-Coulter, Buckinghamshire, United Kingdom) and coverslipped for microscopic analysis. Control cells were incubated with preadsorbed primary antibodies with a specific immunogen blocking peptide.

### 2.7 Ligand stimulation and cAMP assay

Butaprost and/or PGF-induced cAMP accumulation was determined by seeding 2 × 10^5^ Ishikawa cells/well in 6-well plates. The cells were serum-starved in the presence of 3 µg/ml of indomethacin (a dual Cox enzyme inhibitor used to inhibit production of endogenous prostaglandins). Thereafter, the cells were pre-treated with the phosphodiesterase inhibitor IBMX (Sigma) to a final concentration of 0.2 mM in serum-free medium for 30 min. Cells were treated for 5 min with vehicle, Butaprost (5 µM) and/or PGF (100 nM) in the presence/absence of different chemical inhibitors as described in the figure legends. After incubation the cells were lysed in R&D Cell Lysis Buffer™ (R&D Systems, Oxford UK) and cAMP release was determined by ELISA using cAMP Kit (R&D Systems) according to manufacturer's protocol. The concentration of cAMP was calculated using a standard curve by Assay Zap (Biosoft, Cambridge, UK) and was normalized according to the respective protein concentration of each sample. Data are represented as mean ± SEM.

### 2.8 Total inositol phosphate (IP) assay

PGF and/or Butaprost-induced accumulation of IP was determined as previously described [20]. Briefly, Ishikawa cells (50,000 cells/well) were seeded in 24-well plates and allowed to adhere overnight. The following day, cells were labeled with 0.5 µCi/well myo-^3^H-inositol (Amersham Bioscineces, RSA) in an inositol-free DMEM 199 media supplemented with 2% dialyzed fetal calf serum overnight. The next day, cells were stimulated with PGF and/or Butaprost with the required concentration for an hour at 37 °C while non-stimulated samples were taken as control for each experiment. After aspirating the buffer, cells were lysed by the addition of 1 ml ice-cold 10 mM formic acid and the plates were placed on ice for 30 min. Total ^3^H-inositol phosphates were separated from cell extracts on AG 1-X8 resin by anion exchange chromatography and counted by scintillation counting in Liquid Scintillation Analyzer (Packard GmbH, Frankfurt, Germany). Data are represented as mean ± SEM and expressed as fold increase above non-stimulated samples.

### 2.9 Knockdown of AC1 and AC3 with siRNA transfection

Three different Stealth siRNA duplex oligoribonucleotides (Invitrogen, Paisley, UK) were used to abolish the expression and function of AC1 and AC3. FPEP2 Ishikawa cells were seeded (7.5 × 10^4^ cells/well) in complete media into 12-well plates. On the day of the transfection, the cells were exposed to 60 nM (20 nM from each Stealth siRNA) AC isoform-specific or scrambled sequence siRNA in the presence of SuperFect^®^ (QIAGEN, Crawley, UK) for 6 h. After 48 h of transfection the cells were subjected to RNA extraction for AC1 and AC3 mRNA expression analysis or serum-starved overnight in medium containing indomethacin (3 µg/ml) for cAMP assay. For cAMP assay, cells were then exposed to either Butaprost (5 µM) and/or PGF (100 nM) for 5 min, lysed and subjected to cAMP analysis as described earlier.

### 2.10 Gene array and data analysis

Ishikawa FPEP2 cells were seeded at a density of 5 × 10^5^ cells, serum-starved for 18 h in the presence of 3 µg/ml of indomethacin. Cells were then treated in serum-free media for 8 h with vehicle, Butaprost (5 µM) and/or PGF (100 nM). Treatments were performed in triplicate and repeated four times, (total of *n* = 12 for each treatment). After treatment, the cells were washed with ice-cold PBS, total RNA was extracted from each sample, RNA was analyzed and triplicates pooled to produce four samples of each treatment for array analysis. RT-IVT was then carried out in accordance to the Applied Biosystems Chemiluminescent RT-IVT nanoamp (one-cycle) labelling protocol. Samples were fragmented and hybridized to AB1700 version 2 Applied Biosystems Human Genome Survey microarrays. After hybridization the GeneChip arrays were stained and washed on the fluidics station and scanned. Data acquired using ABI technology was pre-processed according to the manufacturers' recommendations. The data was normalized using variance stabilized normalization [21]. Normalized data were analyzed for differential expression with the LIMMA package as described in the LIMMA user guide [22]. The *P* values were adjusted for multiple testing with Benjamini and Hochberg method [23]. The resulting gene list included only the genes that had a fold change value of 2.0 or higher and a *P* of <0.05. Bioinformatics was performed using the gene set analysis tool kit [24]. A hypergeometric test was used to calculate significantly overrepresented ontologies from the gene list. Array hybridization and data analysis was performed by GeneService Ltd (Cambridge, UK). Gene Ontology annotations were assigned to classify Butaprost and/or PGF regulated genes for biological processes and molecular functions using a web tool provided by the gene ontology database (www.geneontology.org).

### 2.11 Statistical analysis

All data are presented as mean ± S.E.M. Statistical significant differences were determined by one-way analysis of variance using Prism 5.0 software (GraphPad Software Inc., San Diego, CA) (*, *P* < 0.05; **, *P* < 0.001; ***, *P* < 0.0001).

## 3 Results

### 3.1 EP2 and FP receptor expression in stably transfected Ishikawa cells

**Fig. 1:**
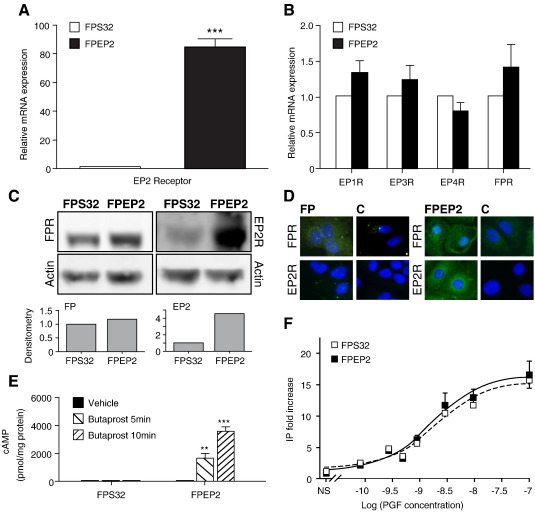
EP2 and FP receptor analysis in FPS32 and FPEP2 cells. Relative mRNA expression of (A) EP2 receptor (B) EP1, EP3, EP4 and FP receptors as determined by quantitative real-time RT-PCR analysis in FPS32 and FPEP2 cells. Expression levels in FPEP2 clones are expressed as fold increase above FPS32 cells (*n* = 4; ***, *P* < 0.0001). (C) Protein expression of FP and EP2 receptors normalized for loading against ß-actin and expressed as fold increase above FPS32 cells as determined by Western blot analysis. (D) Localization of the FP (FPR) and EP2 (EP2R) receptor in FPS32 and FPEP2 cells as determined by immunofluorescence microscopy. Control cells (C) were incubated with preadsorbed primary antibody using specific blocking peptides. (E) The effect of 5 and 10 min treatments with vehicle or Butaprost on cAMP release in FPS32 and FPEP2 cells as determined by cAMP ELISA analysis (*n* = 4; **, *P* < 0.001; ***, *P* < 0.0001). (F) The effect of 60 min PGF stimulation on IP response in FPS32 and FPEP2 cell lines. Data are expressed as fold increase above non-stimulated samples (*n* = 5).

In order to investigate the cross-talk between the EP2 and FP receptors, we created a stable cell line expressing the EP2 and FP receptor in Ishikawa cells (FPEP2 cells). We initially assessed the expression of the E prostanoid receptors (EP1, EP2, EP3 and EP4) and FP receptor in FPEP2 cells in comparison with the parental FPS32 cells. As shown in Fig. 1A, quantitative real-time RT-PCR revealed a significant increase of EP2 receptor expression above the parental FPS32 cells (*P* < 0.0001). There was no significant difference in expression of EP1, EP3, EP4 and the FP receptor between the FPEP2 cell line and the parental FPS32 cell line (Fig. 1B). Western blot analysis (Fig. 1C) and immunofluorescence microscopy (Fig. 1D) confirmed elevated expression of EP2 receptor protein in the FPEP2 cells compared to the FPS32 cells while the FP receptor expression was not altered between the two cell lines.

Activation of the EP2 receptor by Butaprost leads to intracellular accumulation of cAMP [25]. In order to assess the functionality of the EP2 receptor in FPEP2 cells, the ability to generate cAMP was determined by treating the cells with vehicle or Butaprost (5 μM) for 5 and 10 min. As shown in Fig. 1E, treatment of FPEP2 cells with Butaprost for 5 or 10 min increased intracellular cAMP accumulation significantly (*P* < 0.001 and *P* < 0.0001, respectively) in a time-dependent manner compared to vehicle treatment. Butaprost treatment of the FPS32 cell line had no effect on intracellular cAMP accumulation compared to vehicle treatment (Fig. 1E).

The FP receptor is a Gα_q_-coupled receptor, which upon PGF activation leads to an accumulation of intracellular IP [25]. In order to compare the functionality of the FP receptors in the parental FPS32 and FPEP2 cell lines, the cells were subjected to increasing doses of PGF administration (10^− 10^ to 10^− 6^ M) while control samples were left untreated for an hour. As shown in Fig. 1F, PGF treatment of both the parental FPS32 and FPEP2 cells gave a dose-dependent increase in IP release with an *E*_max_ value of 15.7 ± 0.6 and 16.6 ± 2.1 and an EC_50_ value of 2.3 nM ± 0.3 and 1.95 nM ± 0.8 for the FPS32 and FPEP2 cell lines respectively, confirming our observations in Fig. 1B and C, that the levels of FP receptor were similar between the FPS32 and FPEP2 cells.

### 3.2 PGF potentiates Butaprost-stimulated cAMP production via FP receptor-Gα_q_ coupling and PLC activation in FPEP2 Ishikawa cells

**Fig. 2:**
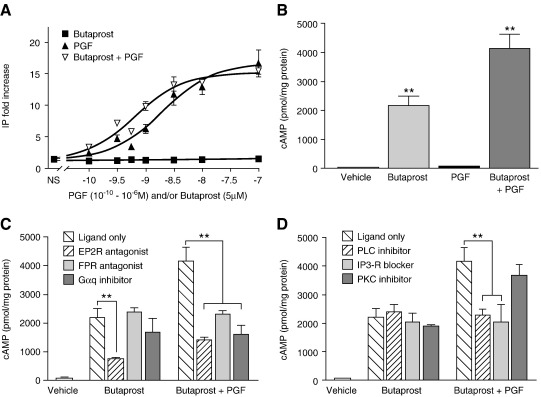
PGF enhances Butaprost-stimulated cAMP production via FP receptor-Gα_q_ coupling and PLC activation. (A) IP release in FPEP2 cells after treatment with PGF (10^− 10^ to 10^− 6^ M) or (5 μM) Butaprost alone or PGF (10^− 10^ to 10^− 6^ M) and Butaprost (5 μM) together for 60 min as determined by an IP assay. Data are expressed as fold increase above non-stimulated sample (*n* = 4). (B) cAMP accumulation in FPEP2 cells after 5 min treatment with vehicle, Butaprost and/or PGF (*n* = 4). (C) cAMP accumulation in FPEP2 cells treated with vehicle, Butaprost and/or PGF in the presence/absence of the EP2 receptor antagonist (AH6809), FP receptor antagonist (AL-8810) or Gα_q_ inhibitor (YM-254890) for 5 min (*n* = 4). (D) cAMP in cells treated with vehicle Butaprost and/or PGF or Butaprost and/or PGF together with the PLC inhibitor (10 µM U73122), IP3-R blocker (40 μm 2-APB) or PKC inhibitor (1 μm Ro-31-822) for 5 min (*n* = 4; **, *P* < 0.001).

We next determined the integrated effect of Butaprost and PGF co-administration on IP and cAMP release. FPEP2 cells were treated with an increasing dose of PGF (10^− 10^ to 10^− 6^ M) or 5 μM Butaprost alone or with PGF (10^− 10^ to 10^− 6^ M) and 5 μM of Butaprost together. As shown in Fig. 2A, Butaprost treatment of the FPEP2 cells had no effect on IP release, since treatment of PGF alone or in combination with Butaprost gave a similar IP response (*E*_max_ 15.5 ± 0.6 and *E*_max_ of 16.6 ± 2.2 and EC_50_ 0.7 nM ± 0.5 and 1.95 ± 1.04, respectively). These data demonstrate that PGF-mediated IP release is not affected by Butaprost treatment. To assess the effect of FP receptor induction by PGF on Butaprost-stimulated cAMP accumulation, FPEP2 cells were treated with vehicle, Butaprost (5 μM) and/or PGF (100 nM) for 5 min. As shown in Fig. 2B, treatment of cells with Butaprost alone gave a robust increase in intracellular cAMP accumulation compared with vehicle treatment (*P* < 0.001), while treatment with PGF alone had minimal effect. However, co-treatment of the cells with Butaprost and PGF together significantly increased the cAMP response compared to treatment with Butaprost alone (Fig. 2B; *P* < 0.001).

To investigate whether the augmentation of cAMP release by PGF is mediated by either the EP2 or FP receptor, FPEP2 cells were treated with Butaprost and/or PGF in the presence/absence of specific antagonist for the EP2 receptor (AH6809; 10 μm) or FP receptor (AL-8810; 50 μm). As shown in Fig. 2C, antagonism of the EP2 receptor significantly decreased (*P* < 0.001) Butaprost-induced cAMP release in both groups (Butaprost only and Butaprost and PGF). In contrast, the FP receptor antagonist only abolished the potentiation of cAMP by PGF without altering basal and Butaprost-stimulated cAMP (*P* < 0.001).

To assess if the PGF potentiation of Butaprost-stimulated cAMP is mediated by FP receptor-Gα_q_ coupling, FPEP2 cells were treated with Gα_q_ inhibitor (YM-254890; 1 μm) in the presence/absence of Butaprost and/or PGF. As shown in Fig. 2C, the use of Gα_q_ inhibitor significantly reduced the level of PGF and Butaprost-stimulated cAMP to the level observed in the Butaprost-treated cells (*P* < 0.001). These data indicate that, PGF potentiation of Butaprost-stimulated cAMP is mediated via the FP receptor activation of Gα_q_.

PGF activated FP receptor-Gα_q_ coupling leads to activation of PLC that results in both IP release, to increase intracellular calcium and DAG release that activates PKC [7,26]. We assessed whether the PGF-mediated enhancement of Butaprost-stimulated cAMP was via the PLC-IP or PLC-PKC pathway. As shown in Fig. 2D, inhibition of PLC using the PLCβ inhibitor U73122 (10 µM) abolished the augmentation shown by PGF demonstrating this Gα_s_–Gα_q_ cross-talk is mediated by PLC activation. In order to determine whether intracellular calcium or PKC activation mediate the observed cross-talk, the cells were incubated with specific IP3R blocker (2-APB; 40 μm) or PKC inhibitor (Ro-31-822; 1 μm) in the presence of Butaprost and/or PGF. Co-treatment of the cells with 2-APB significantly inhibited (*P* < 0.001) the PGF-mediated increase in Butaprost-induced cAMP release but had no effect on Butaprost treatment alone. Whereas inhibition of PKC had minimal effect on reducing the PGF-mediated augmentation of cAMP demonstrating that PGF-mediated increase in cAMP is via the accumulation of intracellular calcium (Fig. 2D).

### 3.3 PGF-induced cAMP potentiation is mediated by intracellular Ca^2+^ transients and activation of the AC3 isoform in FPEP2 Ishikawa cells

**Fig. 3:**
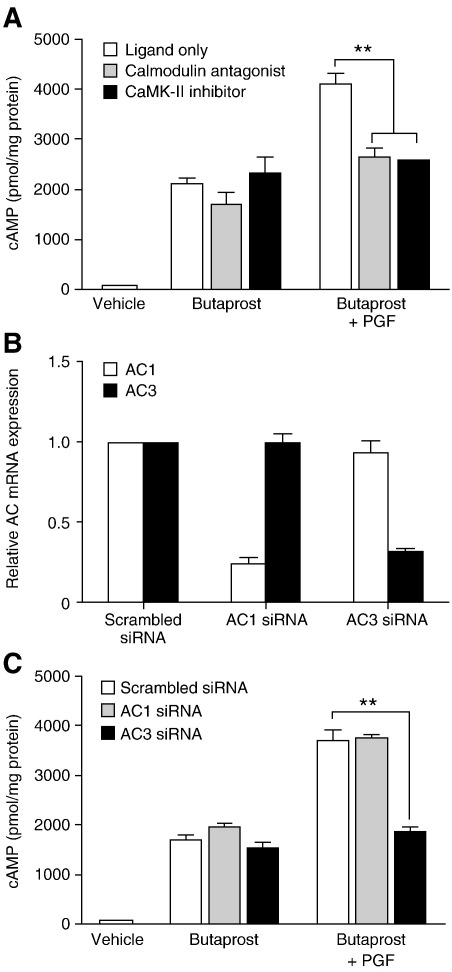
PGF potentiates Butaprost-stimulated cAMP through calmodulin-CaMK-II pathway by activating AC3 isoform in FPEP2 cells. (A) cAMP accumulation in FPEP2 cells after 5 min treatment with vehicle, Butaprost and/or PGF in the presence/absence of inhibitors for calmodulin (W7) or CaMK-II (KN-93) (*n* = 4). (B) Real-Time RT-PCR analysis of AC1 and AC3 in FPEP2 cells transfected with specific AC siRNA compared to the control siRNA (scrambled sequence) (*n* = 4). (C) cAMP accumulation in control, AC1 or AC3 knockdown FPEP2 cells after 5 min treatment with vehicle, Butaprost and/or PGF as determined by cAMP analysis (*n* = 4; **, *P* < 0.001).

Intracellular calcium is known to modulate calcium sensitive isoforms of AC to enhance cAMP production via activation of calmodulin-CaMK-II pathway [27]. To determine whether the PGF-mediated enhancement of cAMP is regulated by the calcium-calmodulin pathway, chemical inhibitors against two calcium effectors were used. Inhibition of calcium pathway, using calmodulin antagonist (W7; 25 μm) or CaMK-II inhibitor (KN-93; 50 μm) significantly reduced (*P* < 0.001) the level of cAMP release seen in FPEP2 cells treated with the combination of Butaprost and PGF to Butaprost-stimulated level (Fig. 3A). These results indicate that PGF potentiation of Butaprost-stimulated cAMP is by the release of Ca^2+^ from intracellular stores leading to activation of calmodulin-CaMK-II pathway.

To investigate which AC isoforms are present in FPEP2 cells, RT-PCR was performed using AC isoform-specific primers. We found that Ishikawa cells express mRNA for AC1, AC3, AC4, AC5, AC6, AC7, AC9 and the soluble AC (SAC), but not AC2 or AC8 isoforms (data not shown). Out of the eight isoforms expressed in Ishikawa cells two of them are known to be calcium stimulated (AC1 and AC3) [11]. Calcium-regulated AC isoforms have been suggested to be involved in Gα_s_–Gα_q_ cross-talk [12–14,28]. To identify which AC isoform is involved in the EP2-FP receptor cross-talk, siRNA designed to AC1 and AC3 were used to knockdown endogenous mRNA expression of the specific isoforms. After specific siRNA transfection into FPEP2 cells, quantitative real-time RT-PCR showed that AC1 and AC3 mRNA expressions were reduced by 76% and 70% respectively compared with scrambled sequence siRNA transfection (Fig. 3B). The specificity of the siRNA was also proven as both AC1 and AC3 siRNA inhibited the expression of their cognate targets without altering the other (Fig. 3B). After 48 h of siRNA transfection, cells were exposed to either Butaprost and/or PGF for 5 min. Thereafter, cells were lysed and subjected to cAMP analysis as described earlier to assess the functional effect of the knockdown. As shown in Fig. 3C, transfection with AC3 siRNA completely abolished the potentiation of Butaprost-stimulated cAMP by PGF significantly (*P* < 0.001) while AC1 siRNA transfection had no effect on cAMP accumulation. This result demonstrates that PGF can enhance Butaprost-stimulated cAMP via the FP receptor-Gα_q_-Ca^2+^-calmodulin pathway by activating the calcium sensitive AC3 isoform.

### 3.4 Gene array analysis

Gene array analysis was used to identify downstream gene transcriptional changes influenced by PGF-enhanced Butaprost-stimulated cAMP signaling. Ishikawa FPEP2 cells were treated with vehicle, Butaprost and/or PGF for 8 h. RNA was extracted, hybridized to AB1700 gene chips, and subjected to gene array analysis. The analysis identified 34 genes whose expression was not regulated by PGF alone, but altered in response to Butaprost and PGF co-treatment compared to treatment with Butaprost alone (Table 2). For example SAT1 gene expression was enhanced from a 5.9 fold increase to a 7.5 fold increase, whereas cytochrome P450, family 26, subfamily A, polypeptide 1 (CYP26A1) gene expression was repressed from an 18.0 fold increase to a 12.3 fold increase. To determine whether the list contained genes with common functions the Gene Ontology database was used to group the genes into functional ontologies. Three functional groups were identified which were represented by 5 or more genes, those for cellular metabolism, immune response and excretion.

### 3.5 Butaprost-mediated SAT1 gene expression is potentiated by Gα_s_–Gα_q_ cross-talk

**Fig. 4:**
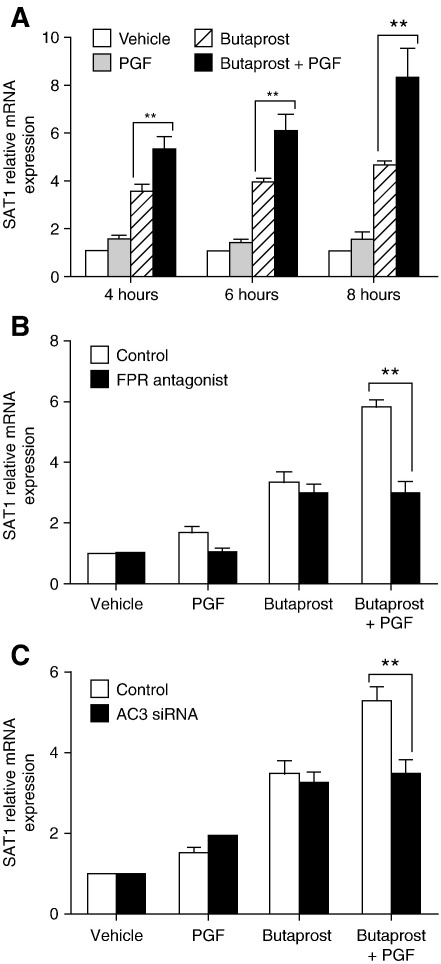
PGF potentiates Butaprost-regulated SAT1 gene expression in FPEP2 cells via the calcium sensitive AC3 isoform. (A) Relative expression of SAT1 in FPEP2 cells after 4, 6 and 8 h treatments with vehicle, Butaprost and/or PGF (*n* = 4). (B) Relative expression of SAT1 in FPEP2 cells after 6 h treatment with vehicle, Butaprost and/or PGF in the absence or presence of the FP receptor antagonist (AL8810) (*n* = 4). (C) Relative expression of SAT1 in FPEP2 cells transfected with siRNA and subsequently treated for 6 h with vehicle, Butaprost and/or PGF (*n* = 4); (**, *P* < 0.001).

Since our array analysis has demonstrated a unique subset of genes, whose expression induced by Butaprost was modulated by PGF, we next investigated the integrated Gα_s_–Gα_q_ cross-talk regulating downstream transcriptional activation by analyzing a candidate gene SAT1, selected from the gene list (Table 2). We focused on the regulation of this gene since it is known to be regulated by cAMP via the cAMP response element binding protein (CREB) that is located on its promoter region [34]. To study the temporal expression of this gene, FPEP2 Ishikawa cells were treated with vehicle, Butaprost and/or PGF for 4, 6 and 8 h. As shown in Fig. 4A, Butaprost treatment alone significantly increased expression of SAT1 at all time points compared to vehicle treatment (*P* < 0.001). No significant elevation of SAT1 gene expression was observed following PGF treatment alone. However, co-stimulation of FPEP2 cells with Butaprost and PGF enhanced the Butaprost-stimulated expression of SAT1 significantly at all time points (*P* < 0.001). To determine whether the PGF-mediated potentiation of SAT1 expression is mediated by the FP receptor, the cells were treated with the FP receptor antagonist (AL8810) in the presence/absence of Butaprost and/or PGF for 6 h. As shown in Fig. 4B, antagonism of the FP receptor completely abolished the potentiation of SAT1 expression by PGF without altering Butaprost-stimulated expression of SAT1. These data demonstrate that Butaprost-regulated expression of SAT1 is augmented by PGF-FP receptor coupling.

Since we have shown that AC3 is involved in the Gα_s_–Gα_q_ cross-talk in FPEP2 cells, we assessed if the same isoform is involved in PGF-mediated potentiation of SAT1 expression. As shown in Fig. 4C, ablation of AC3 expression reduced SAT1 mRNA expression in FPEP2 cells treated with the combination of Butaprost and PGF to the level observed following stimulation with Butaprost alone (*P* < 0.001). These data demonstrate that PGF-mediated potentiation of SAT1 mRNA expression is mediated by the EP2-FP receptor cross-talk via the FP receptor activation of the calcium sensitive AC3 isoform.

## 4 Discussion

There is mounting evidence to support a role for prostaglandins and their respective receptors in endometrial pathologies such as dysmenorrhoea, endometriosis, menorrhagia and endometrial adenocarcinoma [3,5,29,30]*.* Studies in our laboratory and others have used a reductionist approach to dissecting the signaling pathways following the activation of individual EP2 or FP receptors in numerous cell types. We have shown activation of the EP2 and FP receptors can lead to phosphorylation of ERK1/2 via the activation of c-Src and transphosphorylation of the epidermal growth factor receptor (EGFR) in Ishikawa cells and endometrial adenocarcinoma explants ex vivo [7,18]. In addition others have shown activation of the EP2 receptors can trigger the Wnt signaling pathway that involves phosphorylation of glycogen synthase kinase-3 (GSK-3) by Akt to activate β-catenin mediated transcriptional activation in HEK 293 cells [31].

Prostanoid receptors are co-expressed in many cell types [32]. This suggests that co-activation of prostanoid receptors in the same cell could alter the physiological/pathophysiological gene expression profile and outcome. However, to our knowledge there have been no studies addressing the integrative signaling effects of the EP2 and FP receptors on signal transduction and gene expression. Here we show for the first time that co-activation of prostanoid receptors can alter the gene expression profile in endometrial adenocarcinoma cells stably expressing the EP2 and FP receptors. In addition to our knowledge this is the first study to investigate the cross-talk between the EP2 and FP receptors and the molecular mechanism underlying the intracellular signaling pathway in response to co-activation of both receptors.

In order to investigate prostanoid integrative signaling, we stably transfected the EP2 receptor into Ishikawa cells stably expressing the FP receptor. Expression level of the EP2 receptor in FPEP2 cells was compared to the parental FPS32 cells using quantitative real-time RT-PCR, Western blot analysis and immunofluorescence microscopy confirming stable expression of EP2 receptor in FPEP2 cells localised to the perinuclear and plasma membrane. Introduction of the EP2 receptor had no effect on the expression profile of the other E-series receptors and of FP receptor which could be activated by prostanoid ligands in our study. Furthermore, the functionality of EP2 and FP receptors was also confirmed by the release of their respective secondary messengers, cAMP and IP in the presence of their respective ligands.

**Fig. 5:**
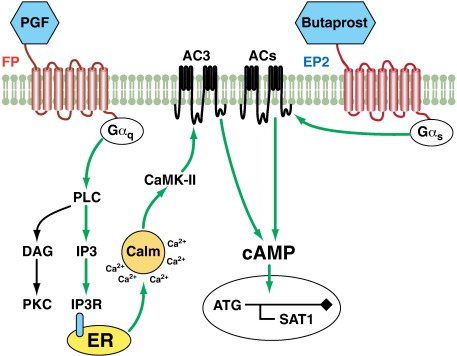
Gα_q_-mediated potentiation of Butaprost-induced cAMP release. Butaprost activates Gα_s_-coupled EP2 receptors resulting in a rapid increase in intracellular cAMP accumulation while PGF by itself does not alter cAMP production. However, co-treatment of the cells with both ligands leads to the Gα_q_-mediated activation of PLC and release of IP3 from the plasma membrane. Subsequently, IP3 via the IP3 receptor (IP3R) mediates the release of Ca^2+^ from intracellular stores leading to calmodulin-CaMK-II dependent potentiation of cAMP release via the calcium sensitive AC3 isoform and modulation of gene transcription such as SAT1.

Using the FPEP2 cells as a model system, we showed that Butaprost stimulation of FPEP2 cells gave a robust intracellular accumulation of cAMP while PGF-mediated FP receptor activation by its own had no effect on cAMP production. Interestingly we found that PGF could significantly enhance the cAMP accumulation in combination with Butaprost. Using specific receptor antagonists and small molecule chemical inhibitors of cell signaling we dissected the signaling pathways mediating the PGF-induced augmentation of cAMP in cells treated with the combination of ligands and have shown that the PGF-enhancement of cAMP observed in FPEP2 cells treated with Butaprost and PGF was induced via the FP receptor-Gα_q_-mediated activation of IP via PLC. Following its release IP could activate its receptors (IPR) present on the endoplasmic reticulum (ER) membrane to promote intracellular calcium release and activation of calmodulin and CaMK-II as depicted schematically in Fig. 5. Numerous studies have shown that the CaMK-II pathway can activate calcium sensitive AC isoforms to regulate intracellular cAMP accumulation [12–14,27,28]. We identified AC1 and AC3 as calcium-regulated targets in our FPEP2 cells by RT-PCR analysis. Transfection studies using siRNA to abolish expression of AC1 or AC3 in FPEP2 cells revealed that the calcium sensitive AC3 (but not AC1) isoform is responsible for PGF-mediated potentiation of Butaprost-stimulated cAMP. These data suggest that the cAMP mediated Gα_s_–Gα_q_ cross-talk reported here is via the activation of the calcium sensitive isoform AC3.

There has been other cAMP mediated Gα_s_–Gα_q_ cross-talk reported but only one with physiological relevance [12–14]. The Gα_s_–Gα_q_ cross-talk reported by Ostram et al. [13] had an important physiological consequence on regulation of the extracellular matrix in myocardium. The authors showed β-adrenergic receptor mediated inhibition of collagen synthesis was further decreased by co-activation of angiotensin II receptor suggesting the cross-talk might play a role in inhibition of fibrosis in heart. In order to determine the integrative effects of receptor co-activation on gene expression, we performed whole genome array profiling in FPEP2 cells in response to Butaprost, PGF or the combination of Butaprost and PGF. Co-activation of FPEP2 cells with Butaprost and PGF enhanced or repressed a set of Butaprost (EP2 receptor) regulated genes. Analysis of the gene list for Gene Ontology annotations indicated functions in cellular metabolism, immune response and excretion. One of the genes identified, SAT1 is an important enzyme in polyamine metabolism, adding an acetyl group to aminopropyl ends of spermidine and spermine [33]. The promoter region of SAT1 lacks TATA box but has multiple binding sites for transcriptional factors including CREB, suggesting it is cAMP regulated [34]. We investigated the integrative signaling mediating the role of prostanoids on SAT1 expression in FPEP2 cells. We found that SAT1 is regulated by the cAMP mediated Gα_s_–Gα_q_ cross-talk via AC3, such that siRNA knockdown of the AC3 isoform completely inhibited the potentiation of Butaprost-stimulated SAT1 expression by PGF. SAT1 is a highly regulated enzyme and it is inducible by polyamines and has been shown to be involved in carcinogenesis [35]. A transgenic increase of SAT1 expression in mice showed a variety of defects such as hair loss, female infertility, impaired lipid metabolism and predisposition to develop pancreatitis [36]. Tucker et al. [37] showed SAT1-over producing transgenic mice bred with Apc^min/+^ mice (mice predisposed to intestinal tumor formation) had an increase in incidence of intestinal tumors while crosses with SAT1 knockout mice led to 75% reduction in tumor load.

Although the role of SAT1 in endometrial pathologies with dysregulated prostanoids is unclear, SAT1 has been shown to have a direct effect on cell migration by binding with α9B1-integrin [38]. Moreover, in a recent study, Vlahakis et al. [39] have demonstrated that α9B1-integrin can bind to the vascular endothelial growth factor (VEGF) to promote angiogenesis. In light of these studies, SAT1 might play a role in pathologies of endometrium by directly promoting cell migration and an indirect enhancement of angiogenesis via α9B1-integrin.

## 5 Conclusions

This study demonstrates that co-activation of the EP2 and FP receptors results in enhanced release of cAMP, in a Gα_q_-calcium-dependent manner via the calcium sensitive AC3 isoform. Activation of this pathway modulates expression of genes involved in metabolism, immune response and excretion. Taken together our data suggest that when both EP2 and FP receptors are co-activated this leads to a unique integrative signaling pathway that modulates downstream gene expression of a subset of EP2 receptor induced genes.

## Figures and Tables

**Table 1:** Taqman primers and probes sequences for EP1, EP2, EP3, EP4, FP receptors, AC1, AC3, SAT1 and 18S.

Target gene		Primer and probe sequence (5′-3′)
EP1 receptor	Forward primer	AGATGGTGGGCCAGCTTGT
	Reverse primer	GCCACCAACACCAGCATTG
	Probe (FAM)	CAGCAGATGCACGACACCACCATG
EP2 receptor	Forward primer	GACCGCTTACCTGCAGCTGTA C
	Reverse primer	TGAAGTTGCAGGCGAGCA
	Probe (FAM)	CCACCCTGCTGCTGCTTCTCATTG TCT
EP3 receptor	Forward primer	GACGGCCATTCAGCTTATGG
	Reverse primer	TTGAAGATCATTTTCAACATCATTATCA
	Probe (FAM)	CTGTCGGTCTGCTGGTCTCCGCTC
EP4 receptor	Forward primer	ACGCCGCCTACTCCTACATG
	Reverse primer	AGAGGACGGTGGCGAGAAT
	Probe (FAM)	ACGCGGGCTTCAGCTCCTTCCT
FP receptor	Forward primer	GCAGCTGCGCTTCTTTCAA
	Reverse primer	CACTGTCATGAAGATTACTGAAAA AAATAC
	Probe (FAM)	CAC AAC CTG CCA GAC GGA AAA CCG
SAT1	Forward primer	CGGGCCGACTGGTGTTTA
	Reverse primer	AGTCAGGCTGGCACCATGAC
	Probe (FAM)	CCGTCACTCGCCGAGGTTCCTTG
AC1	Forward primer	TCTTCGGCAAGTTCGATGAA
	Reverse primer	GCAGTCCCCGAGAATCTTGA
	Probe (FAM)	TAGCCACGGAGAACCACTGTCGCC
AC3	Forward primer	CTGATGTCACTGTAGCCAACAAGA
	Reverse primer	CCACATCAAACTCCCCTTTCA
	Probe (FAM)	CATCCCTGGGCGCGTGCAC
18S	Forward primer	CGGCTACCACATCCAAGGAA
	Reverse primer	GCTGGAATTACCGCGGCT
	Probe (VIC)	TGCTGGCACCAGACTTGCCCTC

**Table 2:** Genes differentially expressed by Butaprost treatment only and modulated in combination with PGF treatment.

Gene symbol	Gene name	Description	Mean fold change	
			Butaprost	PGF and Butaprost
*PGF enhanced modulation of gene expression*				
SAT1	Spermidine/spermine N1-acetyltransferase	Enzyme in the pathway of polyamine metabolism	5.94	7.47
RAPGEF5	Rap guanine nucleotide exchange factor (GEF) 5	GTPase function in signal transduction	3.48	4.05
FRAS1	Fraser syndrome 1	Extracellular matrix protein, adhesion	3.46	6.41
KCNK5	Potassium channel, subfamily K, member 5	Potassium channel	3.20	3.71
ATP1B3	Atpase, Na+/K+ transporting, beta 3 polypeptide	Establishing and maintaining gradients of Na and K ions	3.19	4.28
AQP3	Aquaporin 3	Water channel protein	3.03	3.57
CDC42EP2	CDC42 effector protein 2	Actin filament assembly and cell shape control	3.02	4.16
LIMS3	LIM and senescent cell antigen-like domains 3	Unknown	2.98	3.81
FAM100A	Family with sequence similarity 100, member A	Unknown	2.87	3.55
ZNF323	Zinc finger protein 323	Embryo development	0.16	0.12
NR3C2	Nuclear receptor subfamily 3, group C, member 2	Mineralocorticoid receptor	0.18	0.14
SPAG8	Sperm associated antigen 8	Tumor progression	0.24	0.15
C10orf91	Chromosome 10 open reading frame 91	Unknown	0.27	0.14
DEFB1	Defensin, beta 1	Antimicrobial peptide	0.32	0.29
KIAA1305	Kiaa1305	Unknown	0.32	0.23
ALDH3B2	Aldehyde dehydrogenase 3 family, member B2	Detoxification of aldehydes	0.33	0.29
RNF144B	Ring finger protein 144B	Regulate the stability of p21	0.33	0.27
SYNM	Synemin	Intermediate filament protein	0.34	0.26
MS4A2	Membrane-spanning 4-domains, subfamily A, mem 2	Subunit of the high affinity IgE receptor	0.34	0.29
RLN1	Relaxin 1	Endocrine and autocrine/paracrine hormone	0.35	0.32
OR6W1P	Olfactory receptor, family 6, subfamily W, member 1 pseudogene	Pseudogene	0.35	0.29
TRIM6	Tripartite motif-containing 6	Antiretroviral	0.35	0.30
SAMD13	Sterile alpha motif domain containing 13	Unknown	0.35	0.31

*PGF repressed modulation of gene expression*				
CYP26A1	Cytochrome P450, family 26, subfamily A, polypeptide 1	Regulates the cellular level of retinoic acid	18.03	12.28
RPRM	Reprimo, TP53 dependent G2 arrest mediator	Potential tumor suppressor	11.90	7.76
IL1R2	Interleukin 1 receptor, type II	Receptor that inhibits the activity of its ligands	3.98	3.62
BTBD3	BTB (POZ) domain containing 3	Proliferation and anti-apoptosis	3.30	3.02
ZNF703	Zinc finger protein 703	Repressor of transcription	3.28	3.04
ADA	Adenosine deaminase	Catalyzes the hydrolysis of adenosine to inosine	3.15	2.87
C1orf168	Chromosome 1 open reading frame 168	Unknown	0.23	0.31
DDIT4	DNA-damage-inducible transcript 4	Mediator in RAS-mediated transformation	0.22	0.26
LIPC	Lipase, hepatic	Triglyceride hydrolase and factor for lipoprotein uptake	0.19	0.22
VWA5A	Von Willebrand factor A domain containing 5A	Potential tumor suppressor	0.17	0.23
KCNJ5	Potassium channel, subfamily J, member 5	Potassium channel	0.16	0.22
